# Ketoanalogues of Essential Amino Acids in Chronic Kidney Disease: From Biochemical Rationale to Conditions for Clinical Effectiveness

**DOI:** 10.3390/nu18193157

**Published:** 2026-09-25

**Authors:** Adamasco Cupisti, Claudia D’Alessandro, Vincenzo Panichi, Diego Moriconi, Domenico Giannese

**Affiliations:** Department of Clinical and Experimental Medicine, University of Pisa, Via Roma 67, 56126 Pisa, Italy; claudia.dalessandro@unipi.it (C.D.); vincenzo.panichi@unipi.it (V.P.); diego.moriconi@unipi.it (D.M.); domenico.giannese@phd.unipi.it (D.G.)

**Keywords:** chronic kidney disease, ketoanalogues, ketoacids, essential amino acids, very-low-protein diet, nitrogen balance, metabolic acidosis, protein–energy wasting

## Abstract

Protein restriction is an established nutritional intervention for selected adults with non-dialysis chronic kidney disease (CKD); a supplemented very-low-protein diet (sVLPD) combines marked protein restriction with essential amino acids and their nitrogen-free keto- and hydroxyanalogues (KAs). KAs may accept amino groups and regenerate essential amino acids while limiting exogenous nitrogen, but reversible transamination competes with irreversible oxidative decarboxylation. This narrative review integrates the biochemical pharmacology of KA/EAA mixtures with the clinical conditions required for effectiveness. Controlled and pragmatic trials are contrasted to distinguish efficacy under selected, adherent conditions from effectiveness in routine care. Safe implementation requires achieved protein restriction, adequate non-protein energy intake, correction of persistent metabolic acidosis, metabolic stability, administration with meals, appropriate dosing and structured monitoring; however, the specific effects of these conditions on in vivo KA disposition have not been quantified in advanced CKD. KA/EAA-supplemented VLPD may delay kidney replacement therapy in appropriately selected and supervised patients, but the supplement should be prescribed as part of a complete nutritional strategy rather than as an autonomous drug.

## 1. Introduction

The nutritional treatment of chronic kidney disease (CKD) has several complementary aims, including reducing diet-derived metabolic burden, preserving protein–energy status, controlling electrolyte and mineral abnormalities, supporting cardiovascular and metabolic health, and maintaining quality of life [1,2,3]. This review focuses primarily on two closely related aims: reducing the burden of nitrogenous waste generated by protein catabolism and preserving protein–energy status. As kidney function declines, the capacity to excrete nitrogenous solutes, fixed acids and phosphate is progressively impaired. Reducing dietary protein may therefore ameliorate uremic manifestations and facilitate control of hyperphosphatemia, metabolic acidosis and, depending on food selection, sodium and potassium. Yet protein restriction also narrows the margin for maintaining essential amino acid supply and lean tissue. The central clinical problem is not simply whether protein can be reduced, but how far it can or should be reduced without converting treatment of uremic burden into protein–energy wasting or compromising food-related quality of life, autonomy and social participation [4,5].

Mixtures of essential amino acids (EAAs) and nitrogen-free analogues of EAAs were introduced into the conservative management of advanced CKD in the early 1970s [6,7,8]. An α-ketoacid has the carbon skeleton of the corresponding amino acid but lacks its amino nitrogen (Figure 1). If the ketoacid accepts an amino group by transamination, an EAA is regenerated so that nitrogen is reutilized rather than incorporated into urea. An α-hydroxyanalogue can be oxidized to its ketoacid and enter the same metabolic network (Figure 1). This ‘nitrogen-sparing’ pathway is biochemically valid, but its net contribution is conditional: it depends on whether transamination and amino acid regeneration prevail over irreversible oxidation of the carbon skeleton. Ketoanalogues (KAs) are substrates at a metabolic branch point: they may be transaminated to amino acids, or they may undergo irreversible oxidative decarboxylation and be used as carbon fuel. The nutritional and metabolic context influences which fate predominates [9,10].

Recent guidelines retain a place for KA/EAA-supplemented very-low-protein diet (sVLPD) in selected, metabolically stable adults with non-dialysis CKD. KDOQI proposed a VLPD providing 0.28–0.43 g protein/kg/day with KA/EAA supplementation as one option for adults with CKD stages 3–5 under close supervision [11]. KDIGO 2024 recommends approximately 0.8 g protein/kg/day for adults with CKD G3–G5 in general, while advising that a 0.3–0.4 g/kg/day VLPD supplemented with EAAs or KAs up to a total protein equivalent of about 0.6 g/kg/day may be considered in adults who are willing and able, at risk of kidney failure, and closely supervised [12]. The wording matters: willingness, ability and supervision are part of the intervention, not optional details.

An sVLPD is highly restrictive and difficult to sustain. Its use should therefore be justified by a clinically meaningful, patient-valued advantage over a less restrictive protein intake, balanced against effects on eating enjoyment, social life, workload, cost and pill burden [13]. If the necessary conditions cannot be achieved, a conventional controlled-protein diet that is feasible and acceptable is preferable to an sVLPD that exists only as a prescription.

This narrative review focuses on the biochemical and pharmacological basis of KA/EAA supplementation and, especially, on the conditions required to translate that basis into clinical benefit. The crucial point is that KAs are not autonomous drugs with a fixed effect independent of diet. Their clinical performance is inseparable from achieved protein intake, energy availability, acid–base status, metabolic stability, adherence and monitoring.

### Literature Identification and Scope

This is a narrative, mechanism-focused review rather than a systematic review of the overall efficacy of protein restriction. Relevant literature was identified through PubMed/MEDLINE searches from database inception to 31 August 2026 and by backward reference screening of key articles, evidence syntheses and clinical guidelines. Search concepts and selection priorities are summarized in Table 1. We preferentially considered human CKD studies, metabolic and nitrogen-balance studies, controlled trials, systematic reviews and meta-analyses, clinical guidelines, and selected experimental studies required to explain KA/EAA metabolism. Studies of low-protein diets without KA/EAA supplementation were considered only when needed to define the metabolic or clinical context; the review was not designed to re-evaluate the overall efficacy of dietary protein restriction.

## 2. What Is Being Administered? Amino Acids, Ketoanalogues and Hydroxyanalogues

Nine amino acids are conventionally essential in adults: valine, isoleucine, leucine, lysine, methionine, phenylalanine, threonine, tryptophan and histidine. Tyrosine is normally synthesized from phenylalanine, but this conversion may be impaired in advanced CKD; tyrosine is therefore often regarded as conditionally essential [14]. Not every essential amino acid has a commercially available corresponding ketoanalogue. Methionine is commonly supplied as a nitrogen-free hydroxyanalogue that can be oxidized to the corresponding ketoacid before transamination. Lysine and threonine analogues would be chemically unstable or unsuitable, and analogues of histidine and tryptophan are not generally available. Consequently, the supplements used in the dietary treatment of CKD combine selected KAs with selected EAAs.

Most clinical evidence in CKD concerns the calcium-salt KA/EAA mixture marketed as Ketosteril^®^ (Fresenius Kabi, Bad Homburg, Germany), which contains the calcium salts of the ketoanalogues of isoleucine, leucine, phenylalanine and valine; the calcium salt of the hydroxyanalogue of methionine; and lysine, threonine, tryptophan, histidine and tyrosine [15]. A similar formulation formerly used in Italy (Alfa-Kappa^®^; Kedrion, Barga, Italy) did not include tryptophan. A current Ketosteril^®^ tablet provides 600 mg of KAs/EAAs, approximately 36 mg of nitrogen and 50 mg of calcium (Figure 2); at 14 tablets/day, it contributes 700 mg/day of calcium. Mention of this product is therefore necessary to describe the formulation and exposure used in the evidence base, but it should not be interpreted as evidence of superiority over alternative mixtures. Formulation-independent conclusions remain limited because comparative or head-to-head clinical studies are lacking.

The usual dose studied for an sVLPD is approximately one tablet per 5 kg body weight per day (or 2 tablets/10 kg/day), divided with meals. If a patient cannot safely or consistently achieve the 0.3–0.4 g/kg/day protein target, the preferred first step is a feasible LPD providing approximately 0.55–0.60 g/kg/day rather than a nominal but unachieved sVLPD. KA/EAA supplementation with this LPD should not be automatic. Lower doses, such as one tablet/10 kg/day, have been used, but this approach is empirical, has a weaker evidence base and does not establish an independent kidney benefit of the supplement. It may be considered case by case when indispensable amino acid adequacy or the nutritional safety margin is a concern, provided energy intake and monitoring are adequate. Administration with meals is pharmacologically rational because absorbed carbon skeletons then coincide with dietary amino-group donors and energy substrates. Calcium salts may reduce absorption of tetracyclines, quinolones, iron, fluoride and some other medicines; country-specific product information should therefore be checked and dosing separated when required [15].

At the traditional full dose, a 70 kg patient takes approximately 14 additional tablets each day. This is a substantial burden in advanced CKD, where polypharmacy is common, and may reduce willingness or unintentionally impair adherence. Tablet size and swallowing difficulty are particularly relevant in older or frail patients. Pill burden, dexterity, cognition and swallowing ability should therefore be assessed before treatment and revisited during follow-up.

The mixture of KA/EAA should be regarded as one component of a prescribed metabolic system: protein restriction creates the need and the biochemical opportunity for EAA regeneration, non-protein energy protects the carbon skeleton from oxidation, and the KA/EAA mixture helps preserve indispensable amino acid availability with less nitrogen load.

Ketoanalogues are efficiently absorbed after oral administration. However, the transport pathways of individual keto- and hydroxyanalogues are less well characterized than those of the corresponding amino acids. After absorption, extensive transamination may occur in intestinal tissues, liver, kidney and skeletal muscle, closely linking branched-chain ketoacid disposition to branched-chain amino acid metabolism [16].

## 3. Biochemical Pharmacology of Ketoanalogues: A Reversible Gateway and an Irreversible Exit

Transamination is the first step in the catabolism of many amino acids and is potentially reversible. Aminotransferases transfer an amino group from an amino acid donor, often glutamate, to an α-ketoacid acceptor. For branched-chain substrates, branched-chain amino acid aminotransferase (BCAT) can convert α-ketoisocaproate to leucine, α-keto-β-methylvalerate to isoleucine and α-ketoisovalerate to valine. The same enzyme can catalyze the reverse reaction. Thus, a supplemented BCKA can regenerate its parent BCAA, but the direction and net flux depend on substrate concentrations, tissue compartment and the metabolic state [17,18].

This reaction supplies a biochemical rationale for nitrogen sparing. The amino group incorporated into a newly formed EAA may derive from transamination networks that would otherwise shift nitrogen toward glutamate, ammonia and ultimately urea. In this way, endogenous nitrogen can be reutilized for synthesis of essential amino acids and body proteins. Classic balance studies showed that KA/EAA-supplemented VLPDs can maintain nitrogen balance and induce adaptive conservation of amino acids in selected patients [10,19,20,21]. The concept is therefore not merely theoretical, but its magnitude varies and the advantage is lost when the carbon skeleton is irreversibly oxidized.

Resistance exercise is an important complementary anabolic stimulus: when feasible and appropriately prescribed, it can stimulate muscle protein synthesis and help preserve muscle mass, strength and function in CKD [22,23,24,25]. It should accompany nutritional treatment rather than be viewed as a substitute for adequate energy and amino acid provision. Direct evidence that resistance exercise changes the transamination-versus-oxidation fate of supplemented KAs in advanced CKD is not available.

However, the BCAT reaction competes with an irreversible exit. The branched-chain α-ketoacid dehydrogenase complex (BCKDH) catalyzes oxidative decarboxylation of BCKAs. Its products become acyl-CoA derivatives that enter intermediary metabolism; valine and isoleucine contribute to succinyl-CoA and/or acetyl-CoA, while leucine is ketogenic. Once a BCKA passes through BCKDH, it cannot return to the original BCAA. The carbon skeleton may support ATP production, ketogenesis or gluconeogenic pathways, but its potential to replace the original amino acid has been irreversibly lost [17,18].

Human physiology studies outside CKD reinforce this distinction. In older men, ingestion of BCKAs increased circulating BCAA availability and stimulated myofibrillar protein synthesis, supporting in vivo amination; nevertheless, the response was smaller than that produced by a larger intact milk-protein dose and depended on the nutritional setting [26]. Experimental work indicates that the efficiency with which α-ketoisocaproate substitutes for leucine increases when dietary protein is low [27]. In other words, metabolic need and substrate availability influence the direction: when the corresponding EAA is scarce, conservation and transamination are favored; when amino acids are already abundant or energy is lacking, oxidation becomes more likely.

KAs should therefore not be described as if every administered molecule were quantitatively converted into an EAA, and there is no fixed “conversion percentage” applicable to every patient. Absorption, organ exchange, aminotransferase equilibrium, availability of amino donors and the activation state of oxidative enzymes all have a role.

Importantly, this conditional model has not been quantified in patients with advanced CKD. No stable-isotope, organ-balance or comparable clinical study has directly measured the fraction of an administered KA that undergoes transamination rather than irreversible oxidation under different levels of protein intake, energy balance or serum bicarbonate. Figure 1 and Table 2 should therefore be read as a qualitative, mechanistically based framework supported by balance studies and experimental physiology, not as a quantitative prediction of KA disposition in an individual patient.

Advanced CKD is characterized by altered amino acid profiles [28,29], insulin resistance, inflammation, reduced physical activity and fixed acid retention. These abnormalities are not uniform, but each can modify protein turnover and the fate of KAs. Metabolic acidosis is especially important [30]. Experimental evidence shows that acidosis, rather than azotemia itself, stimulates branched-chain amino acid catabolism and muscle proteolysis [31,32,33,34].

This physiology explains why bicarbonate status cannot be relegated to a safety laboratory item. Chronic metabolic acidosis promotes loss of muscle protein and BCAAs, precisely the substrates that the supplemented diet is intended to preserve. We therefore regard correction of persistent acidosis as a mechanistically justified condition for nutritional safety and for the intended metabolic use of KAs. However, this conclusion is an extrapolation from experimental and clinical evidence on acidosis, proteolysis and BCAA catabolism; clinical studies demonstrating that bicarbonate status modifies the efficacy of KA/EAA supplementation are not available [31,32,33,34,35].

Inflammation and intercurrent illness create a similar problem. Fever, infection, surgery, active malignancy, heart failure and other catabolic states increase amino acid requirements and reduce the safety margin of protein restriction. Insulin deficiency or severe insulin resistance can further favor proteolysis and impair nitrogen balance [36]. In such settings, rigid continuation of an sVLPD may be inappropriate even when supplemented with a KA/EAA mixture; temporary liberalization of protein and energy intake is often more rational. Metabolic stability should therefore be considered a recurring clinical assessment rather than a one-time eligibility criterion.

The kidney also participates in amino acid interorgan exchange and in the metabolism of glutamine, glycine, arginine and phenylalanine/tyrosine [14,37]. Conversely, residual kidney function supports some acid excretion and metabolic flexibility. These observations favor an individualized approach: CKD stage alone does not determine whether a patient can safely implement an sVLPD.

## 4. Conditions Supporting the Intended Metabolic Role of Ketoanalogues

The biochemical framework can be summarized as three linked conditions: a genuine dietary need for EAAs, adequate non-protein energy intake, and control of metabolic acidosis and catabolism. Failure of any one condition may shift the KA supplement away from its intended nitrogen-sparing role and jeopardize nitrogen balance (Figure 1). Figure 1 depicts the competing metabolic pathways, whereas Table 2 translates the conditions favoring either pathway into clinically relevant scenarios.

First, dietary protein and EAA intake must be sufficiently reduced. If abundant high-quality protein already supplies leucine, valine, isoleucine and the other EAAs, there is less physiological need to regenerate them from KAs. Analogues may then be oxidized without conferring a meaningful nitrogen-sparing advantage. This is why KA supplementation cannot be evaluated independently of achieved protein intake. In an sVLPD, EAA provision has deliberately been lowered, and the supplement becomes integral to nutritional adequacy.

Second, energy intake must be sufficient. Current nutritional guidance commonly individualizes energy intake within approximately 25–35 kcal/kg/day according to age, body size, physical activity, obesity and comorbidity [11]. This range is a guideline-based target intended to maintain energy balance and nutritional status; it is not a threshold shown directly to alter KA transamination or clinical efficacy. No clinical trial has randomized energy intake within an sVLPD to test such an interaction. The mechanistic rationale is that, when carbohydrate and fat energy are inadequate, amino acids and KAs are more likely to be oxidized for fuel. The patient may comply with protein restriction yet lose weight and lean tissue because the diet is simply low in energy. Protein-free or very-low-protein special foods can help decouple energy provision from nitrogen, especially in Mediterranean dietary patterns where bread and pasta otherwise contribute substantial protein [38]. Oils, suitable carbohydrate sources and individualized meal planning serve the same metabolic objective. Energy adequacy should be judged longitudinally through weight trajectory, dietary records, appetite and body composition/function rather than inferred from a printed menu or a single calculated target.

The 25–35 kcal/kg/day range refers to total energy intake, not to a prescribed amount of carbohydrate or to “non-protein calories” alone. In practice, the energy remaining after protein-containing foods are limited must be supplied by an individualized combination of carbohydrate and fat. This can be difficult in patients with diabetes, obesity, dyslipidemia, anemia, poor appetite, gastrointestinal symptoms or taste changes: large carbohydrate loads may be metabolically undesirable, whereas added fats may be poorly tolerated or unpalatable. Protein-free staple foods can provide energy with little nitrogen, but their cost, availability, texture and palatability vary and may undermine adherence. The energy prescription must therefore be constructed around glycemic control, comorbidity, appetite, cultural food habits, affordability and patient preference rather than achieved by a uniform high-carbohydrate approach.

Third, persistent metabolic acidosis should be prevented or corrected for established clinical and nutritional reasons [39]. A practical target is to avoid serum bicarbonate below the laboratory lower limit, commonly 22 mmol/L, while recognizing evolving KDIGO guidance and the need to avoid sodium-related harm from indiscriminate alkali therapy [12]. Bicarbonate values should be interpreted in clinical context and repeated when unexpected. Dietary patterns rich in fruits and vegetables can reduce net endogenous acid production, but potassium tolerance must be individualized [40]. Experimental and clinical data indicate that ongoing metabolic acidosis increases muscle catabolism and BCAA oxidation [31,32,33,34,35]; whether correction of acidosis specifically increases in vivo conversion of supplemented BCKAs to their corresponding amino acids in patients with CKD remains untested.

These conditions generate four simplified metabolic scenarios (Table 2). With adequate energy and low EAA availability, transamination and nitrogen reutilization are favored. With adequate energy but abundant EAAs, the need for regeneration is reduced. With energy deficiency, carbon skeletons are increasingly oxidized. When energy deficiency is combined with acidosis or inflammation, the risk of endogenous proteolysis and nutritional deterioration is highest.

## 5. Clinical Evidence for KA/EAA Supplementation: Efficacy, Effectiveness and Adherence

Trials of protein restriction and KA/EAA supplementation span several decades and vary substantially in protein targets, supplements, patient selection, achieved intake and outcome definitions. Meta-analyses generally suggest that supplemented VLPDs can reduce the risk of kidney failure in non-dialysis CKD, while effects on mortality and the independent contribution of KAs remain less certain [41,42,43]. Interpretation is complicated because the intervention is a treatment package. The supplement cannot readily be separated from protein restriction, food pattern, energy provision, dietitian contact and adherence.

Accordingly, available outcome trials do not identify a progressive clinical improvement attributable solely to the KA/EAA tablets. They evaluate the complete supplemented dietary intervention. Evidence more specifically supporting the analogues comes from biochemical and metabolic studies of nitrogen balance, amino acid conservation and in vivo amination [19,20,21,26,27], whereas effects on kidney failure, dialysis initiation and nutritional safety reflect the combined effects of protein restriction, energy provision, food pattern, KA/EAA supplementation, counseling and follow-up. The independent incremental clinical effect of the supplement therefore remains uncertain [44,45].

This review does not aim to reassess the overall efficacy of low-protein diets in CKD, a subject addressed by numerous evidence syntheses. Table 3 therefore includes only selected systematic reviews and meta-analyses needed to interpret KA/EAA supplementation. Across these analyses, the intervention usually combines protein restriction, KA/EAA tablets, dietary counselling and follow-up; consequently, pooled clinical outcomes cannot quantify the independent pharmacological contribution of the KAs.

The randomized trial by Garneata and colleagues provides a useful efficacy model [46]. Of 1413 patients assessed for eligibility, 207 (14.6%) ultimately underwent randomization after eligibility assessment and a run-in phase that required willingness and ability to follow a protein-restricted regimen. The sVLPD reduced the composite risk of dialysis initiation or a >50% decline in eGFR compared with a conventional LPD, without evidence of nutritional deterioration. This marked selection enrichment strengthens inference about biological efficacy when the intervention is feasible and achieved but substantially limits external validity. The findings should not be extrapolated to all patients with CKD G4, and the expected effectiveness in routine practice will be lower when motivation, food access, dietitian support or follow-up capacity are less favorable. An sVLPD is therefore a specialized option for selected patients and experienced centers, rather than a one-size-fits-all treatment.

Baseline serum urea was high in both Garneata groups (median approximately 187 and 213 mg/dL), despite reported protein intakes near 0.6 g/kg/day [46]. This limits generalizability to contemporary patients with a lower uremic burden and raises legitimate questions about baseline intake assessment. However, serum urea also reflects residual kidney function, catabolism, hydration and gastrointestinal bleeding and cannot by itself demonstrate unreliable dietary reporting. The observation reinforces the need to interpret food records together with urinary urea nitrogen, clinical context and longitudinal changes.

The ERIKA trial provides a complementary effectiveness model [47]. In patients already receiving regular tertiary nephrology care, assignment to an sVLPD did not improve kidney or patient survival compared with a standard LPD in the intention-to-treat analysis. Achieved protein intake in the sVLPD arm was approximately 0.60 g/kg/day, while the LPD arm remained near 0.83 g/kg/day. The neutral result should neither be dismissed as mere non-adherence nor interpreted as a direct refutation of the biochemical rationale of a fully achieved sVLPD. Rather, it estimates effectiveness of prescribing this strategy under pragmatic conditions, in which difficulty attaining the protein target is itself a clinically important limitation. The Garneata and ERIKA studies therefore address different questions: efficacy in an adherence-enriched population and effectiveness when the intervention is offered in routine care.

Duration is an additional limitation of the evidence base. The randomized intervention in the Garneata trial lasted 15 months (with eGFR slope assessed after the initial 3-month hemodynamic/dietary adaptation), rather than 12 months, but this remains short relative to the lifelong course of CKD. Existing trials do not establish whether initial adherence, nutritional safety and kidney-replacement-therapy benefits are sustained over many years. Long-term observational experience is informative but remains vulnerable to selection and survivor bias.

The collective evidence supports a deliberately restrained conclusion. The most consistent clinical signal is a reduction or delay in kidney replacement therapy, whereas effects on eGFR slope and the biological rate of kidney-function loss are less consistent [38,48]. Deferrals of dialysis may reflect slower progression, reduced generation of nitrogenous and other uremic solutes that permit conservative management at a lower eGFR, or both. Available trials support nutritional safety in selected, supervised patients, but do not establish a mortality benefit [41,42,43,46,47]. These outcomes should therefore not be combined into a single claim of “renal protection”.

The pivotal dietary trials also largely predated widespread combined use of renin–angiotensin system blockade, SGLT2 inhibitors, finerenone and GLP-1 receptor agonists. No randomized trial has established the absolute incremental benefit of an sVLPD plus KA/EAA on top of current multidrug kidney protection. The rationale for integration remains strong, particularly in advanced CKD because dietary treatment reduces nitrogenous and other diet-derived solute loads that these drugs do not directly address [49,50]; nevertheless, the magnitude of additional benefit in contemporaneously treated patients is uncertain and should not be assumed. Emerging observational evidence suggests that combining KA/EAA-supplemented low-protein diets with SGLT2 inhibitors may be feasible and potentially beneficial, but randomized evidence is lacking [51].

From a patient perspective, sVLPD plus KA/EAA should not compete with or replace evidence-based pharmacological kidney protection. There is no head-to-head data comparing its treatment burden or incremental benefit with SGLT2 inhibitors, finerenone or GLP-1 receptor agonists. Shared decision-making should therefore consider the expected absolute kidney-failure risk, tolerance and eligibility for these drugs, residual uremic burden, treatment workload and the patient’s goals. A supplemented diet may have a particular role when advanced CKD is accompanied by a strong preference to defer dialysis, when standard therapies are not tolerated or are insufficient, or within kidney supportive/conservative care, provided nutritional safety and quality of life remain acceptable.

## 6. Translating Pharmacology of Ketoanalogues into a Clinical Prescription

As emphasized by the concept of the LPD as a medication, nutritional treatment requires an indication, a defined dose, correct administration, monitoring of efficacy and safety, and adjustment or withdrawal when appropriate [52]. Potential candidates are metabolically stable adults with progressive CKD G4–G5 not on dialysis, a meaningful risk of kidney failure, and a preference to pursue conservative nutritional treatment or postpone dialysis. Earlier use may be reasonable in selected patients, but the absolute benefit of an sVLPD is more uncertain when kidney-failure risk is low. Careful clinical judgement is mandatory. The patient must be able to understand the dietary structure, obtain appropriate foods and supplements, attend monitoring visits and report difficulties or unwanted side effects [53].

Relative reasons to avoid or postpone an sVLPD include active protein–energy wasting, recent unintentional weight loss, anorexia, acute catabolic illness, uncontrolled metabolic acidosis, major gastrointestinal disease limiting dietary intake, inability to adhere safely, and circumstances in which dialysis is urgently indicated [52]. Frailty and older age are not automatic exclusions, but they lower tolerance for errors and require particularly close assessment of residual kidney function and dietary intake. In patients with diabetes, glucose control should be optimized, because poorly controlled diabetes represents a state of metabolic instability and may impair adaptation to protein restriction [53,54].

Before starting, record weight history, edema-free or clinically appropriate reference weight, BMI, appetite, dietary habits, functional status, serum bicarbonate, potassium, calcium, phosphate, urea, creatinine/eGFR, albumin and markers of inflammation when indicated. A dietitian-led estimate of usual protein and energy intake is essential. Baseline information should permit subsequent distinction between intended metabolic effects and nutritional deterioration [55].

Protein–energy wasting should be assessed across complementary domains rather than inferred from serum albumin alone. The International Society of Renal Nutrition and Metabolism framework requires abnormalities in at least three of four categories: biochemical indices, low body mass, reduced muscle mass and low dietary intake [56]. Handgrip strength and validated tools such as the Subjective Global Assessment can add functional and clinical information [57,58]. Baseline findings should be used to define patient-specific warning thresholds before the diet is started.

The diet should then be constructed rather than merely quantified. A conventional LPD generally supplies about 0.55–0.60 g/kg/day; an sVLPD supplies about 0.3–0.4 g/kg/day plus one tablet of KA/EAA mixture per 5 kg body weight. Use ideal or adjusted body weight when actual weight would materially distort the prescription, while recognizing that no single standardized calculation is appropriate for every patient [59]. Energy intake is commonly individualized within approximately 25–35 kcal/kg/day. Protein-free specialty foods may facilitate energy provision without increasing nitrogen, phosphorus or potassium intake [38]. The renal dietitian translates the prescription into the patient’s actual meals.

Early follow-up, for example, after 2–4 weeks, is more useful than waiting several months. Four questions are central: Was the protein target achieved? Was energy intake adequate? Was the supplement taken with meals at the intended dose? Has the patient remained metabolically stable? Thereafter, intervals can be individualized, often every 1–3 months, according to CKD severity, nutritional risk and the reliability of adherence.

Monitoring should integrate dietary, anthropometric, functional and biochemical domains [60]. Weight loss, declining intake, falling handgrip strength, worsening function, persistent metabolic acidosis or hyperkalemia, symptomatic uremia and rising calcium require action. A fall in serum urea, interpreted together with 24 h urinary urea excretion when a reliable collection is available, is a useful biochemical indicator that protein restriction has been achieved; it is not, by itself, proof of nutritional safety. Low urea may reflect successful nitrogen restriction or inadequate intake. Conversely, higher-than-expected urea may indicate excess protein, catabolism, dehydration, gastrointestinal bleeding or corticosteroid exposure. Trends and context matter more than a single value.

If the target for sVLPD is not achieved, identification of barriers is needed. They include hidden protein in bread and cereal products, portion size, restaurant meals, supplement pill burden, cost, taste fatigue, family routines and fear of inadequate eating. The correct response may be simplification to a 0.6 g/kg/day LPD, not repeated failure at 0.3 g/kg/day. A less restrictive diet that is understood and achieved can be metabolically superior to an sVLPD that is not correctly implemented. Whether KA/EAA supplementation should accompany the simplified LPD must then be reassessed rather than continued by default, using the decision pathway in Table 4.

The viability of KA/EAA supplementation is also shaped by social determinants of health. Affordability and reimbursement of the tablets and protein-free foods, food security, suitable cooking and storage facilities, health literacy and language, transport, paid work and caregiving demand, and access to renal dietitian and serial laboratory monitoring can determine whether the prescribed metabolic system is actually delivered. In countries or communities where supplements, specialty foods or follow-up are unavailable or unaffordable, a full dose sVLPD may be inequitable, impractical or unsafe. Feasibility should therefore be assessed before prescription and repeatedly thereafter; a simpler controlled-protein diet using locally available foods is preferable when the complete KA/EAA-supported strategy cannot be implemented reliably [52,53].

## 7. Safety, Monitoring and Remaining Uncertainties About KA/EAA Supplementation

The principal nutritional concern is loss of lean body mass and the development or worsening of protein–energy wasting. Available controlled studies indicate that an sVLPD can be nutritionally safe under supervision, but safety is conditional on adequate energy, metabolic stability and monitoring [19,20,21,46,47]. The supplement does not abolish risk. Prespecified reasons for prompt reassessment should include a sustained fall in energy intake, clinically meaningful unintentional loss of edema-free weight (for example, >5% over 3 months or >10% over 6 months), progressive decline in muscle mass or handgrip strength, development of PEW across validated assessment domains, or an intercurrent catabolic illness [56]. No single value should mandate withdrawal in isolation. Confirmed deterioration should trigger correction of reversible causes and usually temporary or permanent liberalization of the diet; persistent deterioration despite intervention is a reason to stop the sVLPD [52].

Calcium exposure is formulation specific. Calcium-salt KA products may contribute several hundred milligrams of elemental calcium daily. Hypercalcemia and positive calcium balance are concerns in advanced CKD, particularly when KA tablets are combined with calcium-based phosphate binders or active vitamin D; long-term vascular effects specifically attributable to KA formulations have not been established. Before and during treatment, total calcium exposure should be reconciled, serum calcium monitored and the need for calcium-based binders, active vitamin D and other calcium supplements reassessed in accordance with CKD–mineral and bone disorder guidance [61]. If hypercalcemia or an unacceptable calcium burden persists and the concomitant treatments cannot safely be modified, the dose or continuation of the calcium-containing KA/EAA product should be reconsidered. Its calcium load may reduce phosphate absorption but should not be regarded as uniformly beneficial [15].

Phosphate control may improve because protein restrictions lower phosphate intake, plant phosphate is less bioavailable, and calcium salts may bind intestinal phosphate. These effects should be measured, not assumed [62].

Because KA/EAA tablets contain phenylalanine or its analogue, they require specialist consideration in disorders of amino acid metabolism, including phenylketonuria. Hypercalcemia and disturbed amino acid metabolism are listed contraindications [15].

Two major KA-specific uncertainties remain. First, the optimal KA/EAA dose with a 0.55–0.60 g/kg/day LPD is less well established than the traditional full dose with an sVLPD. Second, routine clinical markers cannot quantify in vivo transamination versus irreversible oxidation; stable-isotope studies integrating dietary protein, energy balance and acid–base status could help test the conditional metabolic model proposed here.

Since an sVLPD is sometimes referred to as a ‘keto diet’, terminology should be standardized. The term ‘keto diet’ is easily confused with a ketogenic diet and should be avoided. Reports should specify actual protein intake, energy intake, KA/EAA formulation and dose, adherence, bicarbonate status and nutritional outcomes.

## 8. A Practical Framework: The Clinical Checklist for KA/EAA Supplementation

A concise checklist can align the biochemical rationale with bedside practice.

1. Indication: Is the patient at meaningful risk of kidney failure and postponing dialysis or reducing uremic burden a shared goal?
2. Stability: Is the patient free of active catabolic illness and clinically significant protein–energy wasting?
3. Achievability: Has the patient demonstrated willingness, cognitive and practical ability, food access and follow-up capacity?
4. Protein target: Is the intended strategy an LPD or sVLPD, and is intake measured against an explicitly defined reference weight?
5. EAA context: Is dietary protein/EAA intake low enough for the analogue mixture to serve its intended replacement role?
6. Energy: Are non-protein calories sufficient to prevent oxidation of amino acids and KAs for fuel?
7. Acid–base status: Has persistent metabolic acidosis been corrected and is it being monitored?
8. Exposure: Is the correct formulation taken in the correct dose, divided with meals, with attention to pill burden and interactions?
9. Monitoring: Are weight, intake, strength/function, bicarbonate, potassium, calcium, phosphate, urea and kidney trajectory reviewed serially?
10. Exit plan: Are there predefined reasons to liberalize the diet, pause supplementation or initiate kidney replacement therapy?

This checklist also clarifies interpretation of failure. If protein intake remains near 0.8 g/kg/day, lack of benefit does not demonstrate biochemical failure of transamination; it demonstrates failure to deliver the intended treatment package. If weight falls because energy intake is inadequate, the response should not be more KA tablets. If bicarbonate remains low, correction of acidosis is part of the nutritional intervention. If adherence is good but uremic symptoms progress, dialysis should not be delayed solely to preserve the diet strategy.

## 9. Conclusions

Ketoanalogues offer a biochemically plausible means of supplying the carbon skeletons of selected EAAs with minimal exogenous nitrogen. Their potential benefit rests on reversible transamination and nitrogen reutilization, but their fate is not predetermined: irreversible oxidation competes with EAA regeneration. Low dietary protein/EAA availability, adequate energy intake and correction of persistent metabolic acidosis are clinically prudent components of a safe and coherent supplemented diet; their specific quantitative effects on in vivo KA conversion in advanced CKD remain unestablished.

The evidence supports KA/EAA-supplemented VLPDs as an option for selected, motivated and metabolically stable adults with advanced non-dialysis CKD under close nephrologist and renal-dietitian supervision. Divergent trial results are best understood through the distinction between an achieved intervention and a written prescription. KAs should not be added indiscriminately to an unrestricted diet, used to compensate for insufficient calories, or continued through catabolic illness without reassessment. Their correct unit of treatment is the entire nutritional program: patient selection, a feasible protein target, sufficient energy, acid–base control, dosing with meals, adherence support and serial nutritional and biochemical monitoring [63].

## Figures and Tables

**Figure 1:**
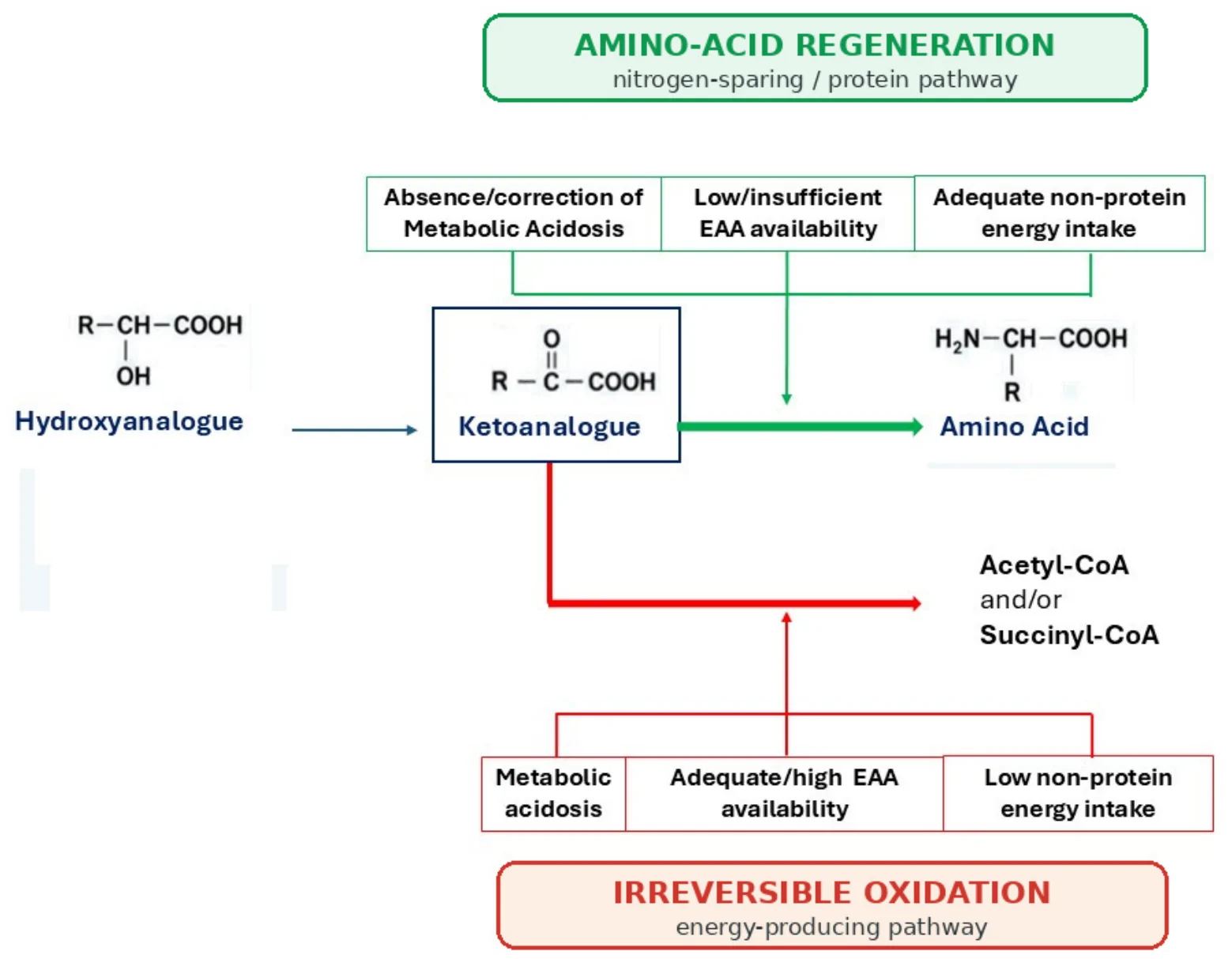
General chemical structures of an amino acid, its α-ketoanalogue and its α-hydroxyanalogue and the proposed conditional metabolic fate of supplemented keto- and hydroxyanalogues. Hydroxyanalogues are oxidized to the corresponding ketoanalogues. Ketoanalogues may undergo reversible transamination to regenerate the corresponding amino acid or irreversible oxidative decarboxylation. Low protein/EAA availability, adequate non-protein energy intake and correction of metabolic acidosis are expected to support their intended nitrogen-sparing role, whereas abundant amino acid availability, inadequate energy intake and metabolic acidosis may increase irreversible oxidation. This scheme is qualitative and has not been quantified in patients with advanced CKD. EAA, essential amino acid. The green pathway identifies amino acid regeneration and nitrogen reutilization; the red pathway identifies irreversible oxidation of the carbon skeleton for energy.

**Figure 2:**
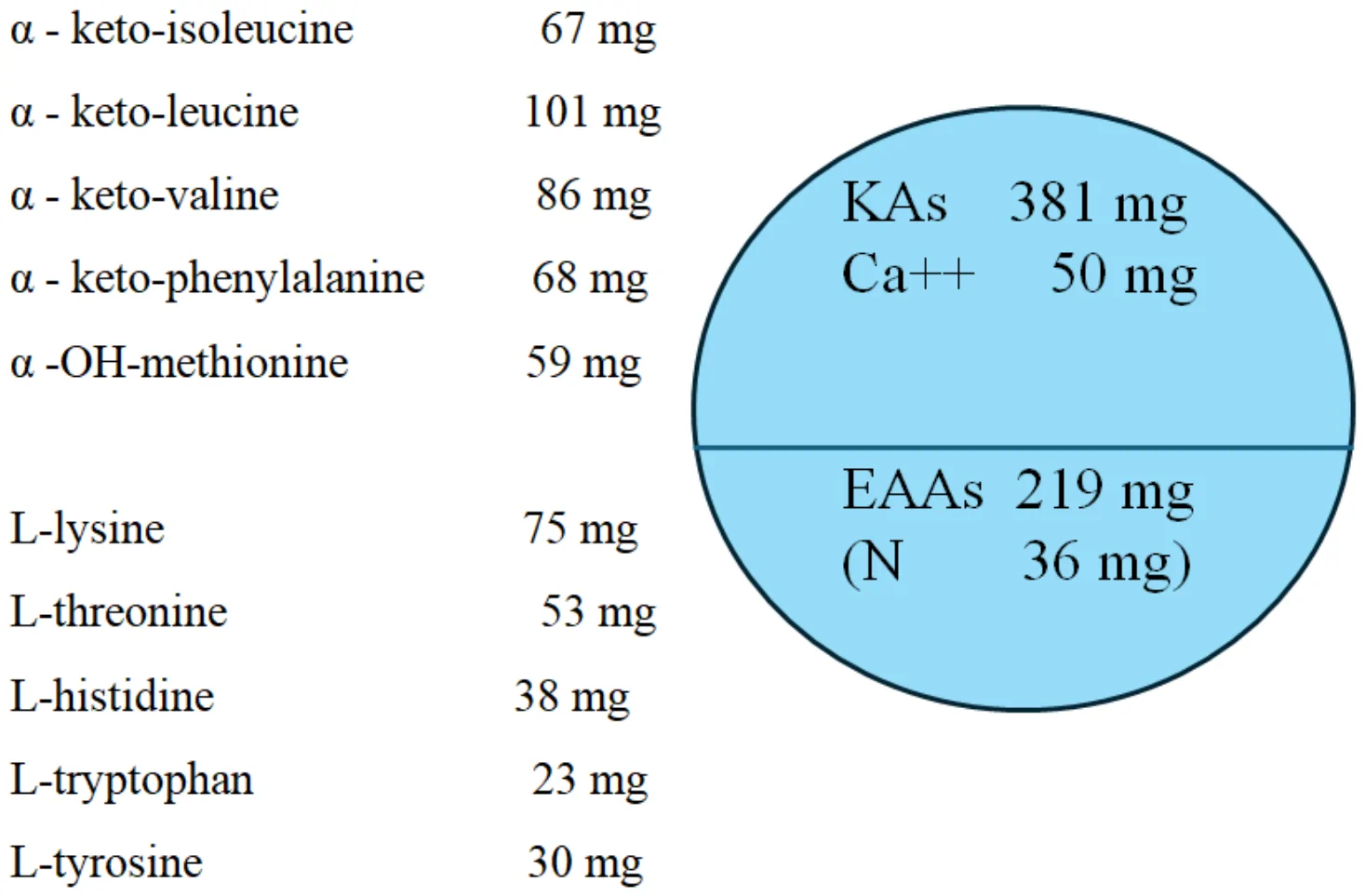
Composition of one tablet of a representative calcium-salt KA/EAA formulation used in CKD. The tablet contains the calcium salts of the ketoanalogues of isoleucine, leucine, phenylalanine and valine; the calcium salt of the hydroxyanalogue of methionine; and the EAAs lysine, threonine, tryptophan and histidine, together with tyrosine. The total KA/EAA mass and the nitrogen and elemental calcium contents are shown. KA, ketoanalogue; EAA, essential amino acid. The composition is presented to define the formulation used in much of the clinical evidence and does not imply superiority over other mixtures.

**Table 1:** Literature identification strategy and scope of the narrative review.

Element	Approach
Bibliographic source	PubMed/MEDLINE; backward screening of key articles, evidence syntheses and clinical guidelines.
Time frame	Database inception to 31 August 2026.
Core search concepts	chronic kidney disease OR renal failure; ketoanalogue* OR keto acid* OR ketoacid* OR essential amino acid*; supplemented low-protein diet OR very-low-protein diet; transamination OR oxidation OR branched-chain metabolism; metabolic acidosis; nitrogen balance; dose; safety; adherence; treatment burden.
Evidence prioritized	Human CKD studies; metabolic and nitrogen-balance studies; controlled and pragmatic trials; KA/EAA-relevant systematic reviews/meta-analyses; guidelines; selected mechanistic experimental studies.
Scope boundary	Low-protein-diet evidence without KA/EAA supplementation was included only when necessary to explain metabolic context, feasibility or interpretation; this was not a systematic reassessment of dietary protein restriction.

**Table 2:** Nutritional context and the expected predominant fate of ketoanalogues. This qualitative model is intended for clinical reasoning; individual conversion and oxidation fractions are not measurable in routine practice.

Dietary/Metabolic Context	Likely Predominant Fate	Clinical Interpretation	Expected Nitrogen-Sparing Usefulness
Low EAA availability + adequate energy + no significant acidosis	Transamination and regeneration of the corresponding EAA are favored	Best setting for nitrogen sparing and preservation of protein synthesis	High: favorable conditions for nitrogen-sparing utilization
Adequate/high EAA availability + adequate energy	Reduced need for EAA regeneration; a larger fraction may be oxidized	Adding KAs to an unrestricted protein intake has weak metabolic rationale	Limited: little metabolic need for EAA regeneration
Adequate/high EAA availability + energy deficit	Oxidation of carbon skeletons for energy	Little nitrogen-sparing benefit; KA tablets do not correct calorie deficiency	Low, until the energy deficit is corrected
Low EAA availability + energy deficit and/or acidosis	Some transamination, but increased irreversible oxidation and endogenous proteolysis	Highest risk of weight and muscle loss; correct energy deficit/acidosis or liberalize diet	Potential, but impaired until energy deficit and/or acidosis is corrected

**Table 3:** Selected evidence syntheses relevant to KA/EAA supplementation and their implications for KA-focused clinical interpretation.

Evidence Synthesis	Intervention Examined	KA/EAA-Relevant Finding	Main Limitation for KA-Specific Inference	Clinical Interpretation
Hahn et al., 2020 [41]	Low- and very-low-protein diets in non-diabetic CKD; supplemented regimens included in the broader dietary evidence.	Very-low-protein strategies may reduce progression to kidney failure, while effects on mortality remain uncertain.	KA/EAA exposure was not uniform, and its incremental contribution could not be separated from protein restriction and dietary care.	Provides context for the complete dietary strategy, not proof of an autonomous KA effect.
Yan et al., 2018 [42]	Different degrees of dietary protein restriction, including supplemented very-low-protein regimens.	More intensive protein restriction showed favorable kidney-outcome signals in pooled analyses.	Heterogeneity in protein targets, supplements, adherence and outcomes limits attribution to KA/EAA.	Achieved diet and co-interventions must be considered when interpreting pooled effects.
Li et al., 2019 [43]	KA supplementation combined with protein-restricted diets.	Pooled findings suggested slower CKD deterioration, particularly in advanced CKD.	Small and heterogeneous studies used different diets, KA doses, follow-up periods and outcome definitions.	Supports a possible benefit of the supplemented strategy but does not establish a fixed KA conversion or treatment effect.
Chen et al., 2024 [44]	KA supplementation plus protein-restricted diets in advanced CKD.	The synthesis supported potential kidney benefits without a consistent signal of nutritional harm under monitored conditions.	The supplement remained embedded within a multidimensional dietary intervention; adherence and achieved protein intake varied.	Most applicable to selected, supervised patients; independent KA efficacy and routine-care effectiveness remain uncertain.

**Table 4:** Practical decision algorithm for KA/EAA supplementation in adults with non-dialysis CKD.

Decision Point	Recommended Clinical Action	KA/EAA Implication
Potential candidate	Confirm progressive CKD G4–G5, meaningful kidney-failure risk and a shared goal of reducing uremic burden or postponing kidney replacement therapy. Exclude active catabolic illness and clinically significant protein–energy wasting.	Proceed only if the patient is metabolically stable and the dietary strategy is feasible.
Can the patient safely and realistically achieve 0.3–0.4 g protein/kg/day?	Yes: construct an individualized sVLPD with adequate energy, renal-dietitian support and access to appropriate foods.	Use the studied full dose, approximately one tablet/5 kg/day, divided with meals, after checking the actual formulation, calcium exposure and interactions.
If the sVLPD target cannot be achieved	Prescribe a feasible LPD providing approximately 0.55–0.60 g protein/kg/day rather than maintaining a nominal but unachieved sVLPD.	Do not continue or initiate KA/EAA automatically. Case-by-case use at a lower empirical dose, often one tablet/10 kg/day, may be considered when amino acid adequacy or nutritional safety is a concern; independent kidney benefit is unproven.
Before initiation	Record reference weight, recent weight trajectory, appetite, intake, strength/function, bicarbonate, potassium, calcium, phosphate, urea and kidney trajectory; reconcile calcium/vitamin D and interacting medicines.	Define formulation, dose, timing with meals, treatment burden and patient-specific monitoring or stopping criteria.
Early review, usually 2–4 weeks	Check achieved protein and energy intake, weight, tolerance, pill exposure, biochemical variables and uremic symptoms; address practical barriers.	Continue only if the prescribed diet and supplement are actually being taken and nutritional safety is maintained.
Ongoing review, often every 1–3 months	Trend intake, edema-free weight, appetite, handgrip/function, bicarbonate, potassium, calcium, phosphate, urea and kidney trajectory; reassess goals and feasibility.	Adjust diet or dose according to achieved intake, tolerance and clinical course; do not use additional tablets to compensate for inadequate energy.
Reduce, pause or discontinue	Act on catabolic illness, progressive weight or functional loss, inadequate energy, refractory acidosis or hyperkalemia, hypercalcemia, intolerable burden, inability to monitor safely, or an indication for kidney replacement therapy.	Liberalize protein intake when required for safety and reduce or stop KA/EAA when the supplemented strategy is no longer feasible, tolerated or clinically appropriate.

## Data Availability

No new data were created or analyzed in this study. Data sharing is not applicable to this article.
